# Evaluation of methicillin-resistant *Staphylococcus aureus* nasal swab screening at a large comprehensive cancer center

**DOI:** 10.1017/ash.2024.370

**Published:** 2024-09-10

**Authors:** Mark J. Herrington, Chun Feng, Hyunsoo Hwang, Nancy N. Vuong

**Affiliations:** 1 Division of Pharmacy, The University of Texas MD Anderson Cancer Center, Houston, TX, USA; 2 Department of Pharmacy Medication Management & Analytics, Division of Pharmacy, University of Texas MD Anderson Cancer Center, Houston, TX, USA; 3 Department of Biostatistics, Research Biostatistician, The University of Texas MD Anderson Cancer Center, Houston, TX, USA

## Abstract

**Objective:**

The aim of this study is to determine the predictive values of MRSA swab screenings in patients with cancer.

**Methods:**

This is a retrospective cohort observational study of adult patients admitted to The University of Texas MD Anderson Cancer Center between January 2019 and October 2022. Data collected from patients with documented MRSA nasal swab screenings and clinical cultures taken within 7 days were collected. The first documented MRSA swab screening and culture results from unique patients were included for analysis to calculate sensitivity, specificity, positive predictive value, and NPV.

**Results:**

A total of 6475 patients with MRSA nasal swab cultures had 13129 clinical cultures from different anatomical sites. Of the patients included, 57% had a solid tumor and 37% had a hematological malignancy, with 82% of patients receiving an anti-MRSA antibiotic prior to MRSA nasal swab. There were 167 documented positive MRSA cultures, most commonly from a wound (41.3%) or respiratory source (24%). Overall sensitivity and specificity for all culture sites were 50.9% and 98.4%, respectively, with an overall NPV of 99.4%. The NPV was 99.8% for bloodstream infections, 98.5% for respiratory infections, 92.6% for wound infections, and greater than 99% for other culture sites.

**Conclusion:**

The specificity and negative predictive value of MRSA swab screenings in patients with cancer was high overall and consistent with the literature in immunocompetent patients. These results may aid in antimicrobial stewardship activities that can help guide the discontinuation of empiric antibiotics in patients with cancer.

## Background

*Staphylococcus aureus* is often found within the nares, throat, axillae, rectum, and groin of patients in both the community and hospital. In the general population, up to 20% are persistently colonized with *S. aureus* while 60% of people are intermittent carriers of S*. aureus*.^1^ Methicillin-resistant *Staphylococcus aureus* (MRSA) is a common cause of hospital and ventilator-associated pneumonia, skin and soft tissue infections, and bloodstream infections.^2,3^ In addition, patients admitted to the hospital often receive empiric broad-spectrum antibiotics until culture results are available, which may take up to 96 hours. Approximately 50–60% of patients receive at least one antimicrobial agent during hospitalization, with vancomycin being one of the most prescribed antibiotics.^4^ Consequently, unnecessary exposure to broad-spectrum antibiotics while awaiting culture results increases the risk of developing antimicrobial resistance, infections with *Clostridioides difficile*, adverse drug effects (eg, nephrotoxicity), drug-drug interactions, extended intensive care unit and hospital length of stay, and increased expense to the health-care system and patient.

Febrile neutropenia remains an important complication of treatment with cytotoxic chemotherapy and is often the first sign of infection in this patient population.^5^ Current guidelines from the Infectious Diseases Society of America and the National Comprehensive Cancer Network recommend empiric antimicrobial therapy against MRSA in patients with IV catheter-related infections, blood cultures positive for gram-positive bacteria, known colonization with MRSA, clinical instability, and soft tissue infections.^6,7^ Empiric vancomycin therapy should be reassessed within 2 to 3 days of initiation. If a resistant gram-positive organism, such as MRSA is not identified, the guidelines recommend discontinuing the vancomycin. However, it is recommended to continue appropriate antibiotics for at least the duration of neutropenia, which often leads to prolonged and unnecessary anti-MRSA therapy while awaiting culture results. A rapid, noninvasive, and inexpensive procedure, with a high negative predictive value (NPV) such as MRSA nasal swab screening, would expedite the de-escalation of empiric antibiotics. The high NPV of nasal swab screening is used in immunocompetent patients to rule out MRSA infections, but limited data exists in the immunocompromised population.

Positive predictive (PPV) values do not correlate well with positive MRSA clinical cultures and therefore may not be confirmatory for MRSA and the ability to target antimicrobial therapy. MRSA nasal swab screening by polymerase chain reaction (PCR) and culture have a consistently high (>95%) negative predictive value (NPV) in ruling out MRSA pneumonia, but the evidence for other infections is variable.^8^ Screening by either PCR or nasal swab cultures has a high concordance rate. However, screening by PCR is more likely to be positive in the setting of concurrent antibiotic administration with positive MRSA cultures as the PCR can detect DNA from nonviable organisms.^9^ The data around the utility of using MRSA nasal swab screening to determine the negative and positive predictive value compared to clinical cultures is lacking in immunocompromised patients. Two retrospective studies suggest a negative MRSA nasal swab may be utilized as an antimicrobial stewardship tool to de-escalate therapy in patients with leukemia. The studies suggest empiric use of anti-MRSA therapy in the acute myeloid leukemia (AML) population may not be warranted given the low prevalence of MRSA pneumonia.^10,11^ A nationwide study conducted across Veteran Affairs (VA) medical centers, evaluated the NPV value beyond respiratory cultures and found high NPVs for a variety of anatomical sites in immunocompetent patients.^12^ Outside the immunocompetent population, the data is limited regarding the evaluation of NPV and PPV at different culture sites in the immunocompromised population. Thus, this study aimed to determine the predictive values of MRSA nasal swab culture in patients with cancer.

## Methods

We conducted a retrospective cohort observational study of all adult (≥ 18 years old) patients admitted to The University of Texas MD Anderson Cancer Center between January 1, 2019 through October 31, 2022. Patients were eligible for study inclusion if they had at least one result from a MRSA nasal swab culture screening and had clinical cultures obtained within 7 days after the MRSA nasal swab culture was ordered. While patients’ subsequent hospital admissions during the study period were excluded from the analysis. Clinical cultures (blood, body fluid, respiratory, wound, and cerebrospinal fluid (CSF)) were included from the first hospital encounter. Patients could contribute to more than 1 clinical culture; however, only the first documented MRSA nasal swab and the first clinical culture from each source were included for analysis to calculate sensitivity, specificity, positive, and negative predictive values.

## Statistical analysis

Baseline characteristics were summarized using descriptive statistics, frequency (%) for categorical variables, and median (25%, 75%) for continuous variables. To evaluate the use of MRSA nasal swabs for the prediction of MRSA in clinical culture, sensitivity, specificity, PPV, NPV, and their associated 95% confidence interval (CI) were calculated. All analyses were conducted in R, version 4.2.1.

## Results

**Figure 1.**
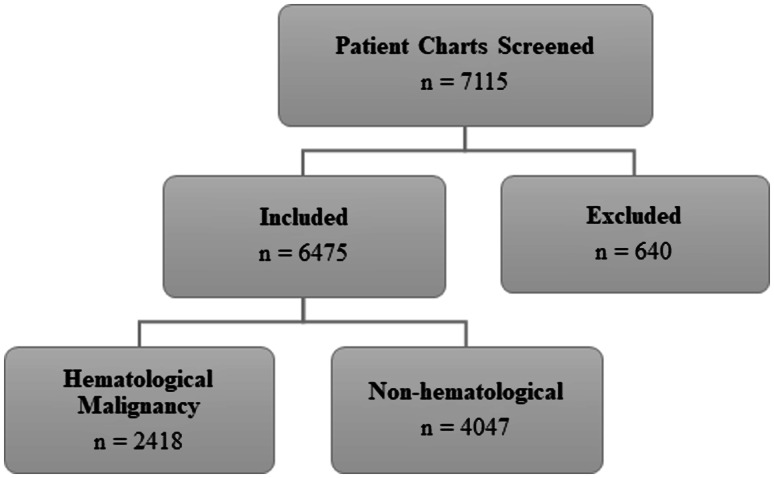
Patient flow diagram.

Of the 7116 patients screened, 6475 patients were included in this analysis (Figure 1). In total, 13129 clinical cultures were yielded from different anatomical sites. Baseline demographic and clinical characteristics are outlined in Table 1. The median age was 65 years (IQR 54–72 years) with most of the population (56.7%) being male. Of the patients included, 57% were admitted to a solid tumor service, 17% to leukemia, 12.6% to lymphoma/myeloma, and 7.8% to the stem cell transplant service. Overall, 82% of patients received an anti-MRSA antibiotic prior to MRSA nasal swab culture. The median hospital length of stay was 9 days [IQR 5–19 days] while the median time to MRSA nasal swab culture result was 2 days. Clinical, cultures consisted of blood [n = 6,137 (46.4%)], urine [n = 4238 (32.3%)], respiratory [n = 1181(8.9%)], body fluid [n = 838 (6.3%)], wound [n = 522 (3.9%), and CSF [n = 213 (1.6%)] as shown in Table 2. Of the patients included, only 294 patients (4.5%) had a positive MRSA nasal swab culture. There were 167 documented positive MRSA cultures, most commonly from a wound [n = 69 (41.3%)] or respiratory source [n = 40 (24%)] (Table 2).

**Table 1.** Baseline characteristics (*n* = 6475)

Characteristic	Total (*n* = 6475)
Median age (IQR)—yr.	65 (54, 72)
Male sex—no. (%)	3668 (56.7)
Median weight (IQR)—Kg	77 (64, 91)
Median height (IQR)—in	67 (64, 70)
Race—no. (%)	
White/Caucasian	4586 (70.9)
Black /African American	836 (12.9)
Asian	365 (5.6)
Other	683 (10)
Admitting Service—no. (%)	
Leukemia	1099 (17)
Stem Cell Transplant	504 (7.8)
Lymphoma/myeloma	815 (12.6)
Solid Tumor	3704 (57.3)
Other	343 (5.3)
Median length of stay (IQR)—Days	9 (5, 19)
Median time to MRSA nasal swab result (IQR)—Days	2 (2, 2)
MRSA treatment prior to MRSA swab—no. (%)	5312 (82)

Note. MRSA, methicillin-resistant *Staphylococcus aureus*; IQR, interquartile range; no., Number.

**Table 2.** Efficacy of methicillin-resistant *Staphylococcus aureus* nares screening by culture site

Type	Number	Negative culture, negative swab (n)	Positive culture, positive swab (n)	Positive culture, negative swab (n)	Negative culture, positive swab (n)	Sensitivity,% (95% CI)	Specificity,% (95% CI)	PPV, % (95% CI)	NPV, % (95% CI)
Total	13129	12753	85	82	209	50.9 (43.6% to 58.7%)	98.4 (98.2% to 98.6%)	28.9 (23.8% to 34.5%)	99.4 (99.2% to 99.5%)
Blood	6137	6011	13	10	103	56.5 (34.5% to 76.8%)	98.3 (97.9% to 98.6%)	11.2 (6.1% to 18.4%)	99.8 (99.7% to 99.9%)
Wound	522	448	33	36	5	47.8 (35.7% to 60.2%)	98.9 (97.4% to 99.6%)	86.8 (71.9% to 95.6%)	92.6 (89.6% to 94.7%)
Urine	4238	4143	11	13	71	45.8 (25.6% to 67.2%)	98.3 (97.9% to 98.7%)	13.4 (6.9% to 22.7%)	99.7 (99.5% to 99.8%)
Respiratory	1181	1126	23	17	15	57.5 (40.9% to 72.9%)	98.7 (97.8% to 99.3%)	60.5 (43.4% to 75.9%)	98.5 (97.6% to 99.1%)
Body fluid	838	818	5	6	9	45.5 (16.6% to 76.6%)	98.9 (97.9% to 99.5%)	35.7 (12.8% to 64.9%	99.3 (98.4% to 99.7%)
CSF/Bone marrow	213	207	0	0	6	n/a	n/a	n/a	n/a

Note. CI, confidence interval; PPV, positive predictive value; NPV, negative predictive value; CSF, cerebrospinal fluid.

Overall sensitivity and specificity for all culture sites were 50.9% and 98.4%, respectively, with an overall NPV of 99.4% and PPV of 28.8% (Table 2). MRSA nasal swab cultures demonstrated a high NPV of 99.8% for ruling out MRSA in blood cultures, which included line-related cultures. Respiratory cultures included bronchoalveolar lavage, sputum, tracheal aspirate, and upper respiratory cultures. The MRSA nasal swab culture produced a high NPV of 98.5% for respiratory cultures. Wound cultures were included from a variety of different culture sites, for which the MRSA nasal swab culture produced a NPV of 92.6%. Body fluid cultures included interventional radiology and intra-abdominal cultures. MRSA colonization identified by MRSA nasal swab culture produced a high NPV of 99.3% for intra-abdominal infections. MRSA nasal swab culture also demonstrated a high NPV of 99.7% for urinary cultures. There were no positive MRSA cultures reported in the CSF.

## Discussion

In this large retrospective study of immunocompromised patients, we showed that negative MRSA nasal swab culture results had consistently high NPV for MRSA infection, regardless of the culture site. These findings support the results from Perreault and colleagues, who retrospectively evaluated the NPV of MRSA nasal swab screenings using PCR and/or culture in 194 AML patients with neutropenic fever.^10^ Their results showed an overall sensitivity of 62%, specificity of 98%, PPV of 38%, and a NPV of 99%. Similarly, the retrospective study by Talagtag and colleagues evaluated 98 AML patients with pneumonia and demonstrated a sensitivity of 75%, specificity of 100%, PPV of 100%, and a NPV of 98.9% using MRSA nasal swab screening.^11^ The high NPV for respiratory cultures is consistent with our study, which showed a NPV of 98.5% and specificity of 98.7% for the utility of MRSA swab screening for detecting MRSA in a clinical culture.

In immunocompetent patients, the literature on the NPV of MRSA nasal swab screenings is predominantly focused on pneumonia, although there is some data from other sources as well.^13^ A meta-analysis by Parente and colleagues included 5163 patients with pneumonia found a NPV of 96.5% with a 10% prevalence of potential MRSA pneumonia.^8^ A large retrospective study conducted by Mergenhagen and colleagues reported an overall specificity of 81.2% and NPV of 96.5% for MRSA nasal screening using PCR and/or culture for any clinical culture site.^12^ The NPV for bloodstream infections was 96.5%, for intra-abdominal cultures 98.6%, for respiratory cultures 96.1%, for wound cultures 93.1%, and for cultures from the urinary system 99.2%. Their NPV for wound and urine cultures (93.1% and 99.2%) were similar to our study (92.6% and 99.7%). Another retrospective study by Noeldner and colleagues evaluated 1989 immunocompetent patients and yielded a NPV of 99.8% for blood, and 92.7% for bone and soft-tissue cultures using PCR assay.^14^ Overall, our current study had higher NPVs compared to the current literature in the immunocompetent population.^13^ The lower MRSA infection rate at our institution and the fact that the majority of cultures obtained were from blood or urine sources were major contributors to the NPV. Additionally, immunocompromised patients often have many cultures drawn at hospital admission due to conditions such as neutropenic and tumor fever. This could result in a falsely elevated NPV; however, we were able to minimize this by only analyzing the first culture taken at each culture site during the hospital admission.

Patients with active cancer carry an even greater risk of infection due to immune suppression from chemo-radiation and frequent healthcare exposure, making it important to understand local MRSA prevalence and epidemiology when deciding on antibiotic therapy. A meta-analysis by Li and colleagues evaluated the pooled prevalence of MRSA bacteremia in cancer patients and showed the prevalence of MRSA among bloodstream infections was 1% in the Americas.^15^ Similarly, studies evaluating MRSA nasal swab screenings found a low prevalence rate of MRSA ranging from 4.1% to 5% among patients with febrile neutropenia and AML.^10,11^ In our study, we had a lower prevalence rate of MRSA infections at 2.6%. Despite the low prevalence rate of MRSA infections in our patient population, the use of empiric anti-MRSA antibiotics remains high. In our study, 82% of patients received at least one anti-MRSA agent prior to MRSA nasal swab culture. The routine use of nasal swab screening may be useful for more rapid de-escalation and avoidance of long-term empirical anti-MRSA therapy. However, delaying anti-MRSA therapy de-escalation is much more common in immunocompromised patients due to the increased risk of organisms such as *Streptococcus* spp, *Enterococcus* spp, and coagulase-negative *Staphylococci*.

This study is the largest to date analyzing the predictive values of nasal swab culture screenings in the cancer population. Our study included a large number of patients and cultures, which increased the validity of the predictive value results. Limitations of our study include the retrospective study design potentially introducing confounding variables resulting from missing or incomplete data. Additionally, only the first hospital encounter per patient was included for analysis, which impacted the total number of clinical cultures and could have excluded positive MRSA cultures. Positive MRSA cultures were based on microbiology culture findings only, which could make it difficult to interpret a true infection versus colonization in respiratory and wound cultures. This limitation could potentially explain the higher PPV seen with the wound and respiratory clinical cultures. Furthermore, 82% of the patients received anti-MRSA therapy before the MRSA nasal swab culture. Prior MRSA therapy may reduce the sensitivity of the MRSA nasal swabs culture however, when antibiotics are administered within the first 48 hours, the reduction in sensitivity is limited.^9^ Lastly, we did not determine if the patients underwent decolonization which would impact the nasal swab culture results.

In conclusion, this retrospective study in patients with cancer showed a high specificity and negative predictive value of MRSA swab culture screenings, which is consistent with current literature for both immunocompromised and immunocompetent patients. These results may aid in antimicrobial stewardship activities and help guide the early discontinuation of empiric anti-MRSA antibiotics in patients with cancer.
